# Exclusive breastfeeding cessation and associated factors among employed mothers in Dukem town, Central Ethiopia

**DOI:** 10.1186/s13006-019-0250-9

**Published:** 2020-02-04

**Authors:** Tolossa Kebede, Kifle Woldemichael, Habtemu Jarso, Bayu Begashaw Bekele

**Affiliations:** 1 Public Health Emergency Management and Health Research Office, Oromia Regional Health Bureau Addis Ababa, Addis Ababa, Ethiopia; 2 Department of Epidemiology, Institute of Health Sciences, Jimma University, 378, Jireen Street, Jimma, Ethiopia; 3 Department of Public Health, College of Health Sciences, Mizan Tepi University, Mizan Aman Street, 260, Mizan Aman, Ethiopia; 4 Doctoral School of Health Sciences, University of Debrecen, Debrecen, 4028 Hungary

**Keywords:** Employed women, Exclusive breastfeeding, Cessation, Dukem town, Ethiopia

## Abstract

**Background:**

Employed women tend to exclusively breastfeed less than non-employed women. Early returning to work has been major reason why employed women stop exclusive breastfeeding. The aim of this study was to investigate exclusive breastfeeding (EBF) cessation and associated factors among employed mothers in Dukem town, Central Ethiopia.

**Methods:**

A cross-sectional study was conducted from February to March 2015 using total sample of 313 randomly selected permanently employed women. Information regarding participants’ work-related factors, health service and sociodemographic factors were collected by face to face interview using structured questionnaire. Data were checked for completeness, entered and analyzed by SPSS version 20. Binary logistic regression was done to identify factors associated with exclusive breastfeeding cessation. The strength of association was measured using odds ratio with 95% confidence intervals.

**Results:**

Prevalence of exclusive breastfeeding cessation was 75.7% (95% CI 71.0, 80.5%). Having a short duration of maternity leave (AOR 9.3; 95% CI 3.8, 23), being a full time employee (AOR 3.5; 95% CI 1.7, 11), being private organization employee (AOR=2.1, 95% CI(1, 4.3)), lack of flexible work time (AOR 3.0; 95% CI 1.2, 7.5), not pumping breast milk (AOR 4.3; 95% CI 1.7, 11), lack of a lactation break (AOR 6.7; 95% CI 3,14.5**)** and work place far away from her child (AOR 3.1; 95% CI 3.1, 6.3), were significantly associated with cessation of EBF among employed mothers.

**Conclusion:**

Prevalence of exclusive breastfeeding cessation was much higher than the international and national expectation. The concerned governmental bodies should consider improving the legislation of the 3 months postpartum maternity leave to reduce employed mother’s exclusive breastfeeding cessation.

## Background

Breastfeeding is the most important part of maternal and child health [1, 2]. According to World Health Organization (WHO) recommendation, 6 months of exclusive breastfeeding (EBF) and continuing breastfeeding up to 2 years and beyond is crucial. In the first 6 months, mother’s milk supplies the best source of nutrition for her child and creates a bond between a child and mother [2]. However, in 2013 globally only 32.07% infants were exclusively breastfed until 6 months of age [3].

Exclusive breastfeeding protects against common and widespread childhood illness such as diarrhea and pneumonia. It may also have longer-term benefits such as lowering mean blood pressure and cholesterol and reducing the prevalence of obesity and type-2 diabetes. Breastfeeding also contributes to the health of mothers; it helps to space children, reduces the risk of ovarian and breast cancer, and the risk of fatal postpartum hemorrhage [1, 4, 5].

Additionally, EBF contributes to a delay in the return of fertility and helps to protect women against anemia by conserving iron. Breastfeeding provides frequent interaction between mother and infant, fostering emotional bonds, a sense of security, and stimulus to the baby’s developing brain [6]. In other words, the cessation of EBF would result in morbidity, mortality and disability. For instance; poor growth, crying and poor sleeping, stunting and poor cognitive ability have resulted from the cessation of EBF [4, 7].

Nevertheless, EBF has some correlates which affect both the health of child and mother. One of these factors is maternal employment. According to *Vanessa M. Oddo* and colleagues, particularly in low and middle-income countries (LMICs), maternal employment is the main reason for termination of EBF among employed mothers [8]. Also, studies from Brazil [9, 10], Ecuador [11], Ghana [12], Kenya [13], and Democratic Republic of Congo [14] concluded that the short duration of the maternal leave is highly correlated with cessation of EBF among employed women than housewives [6, 12]. The identified reason for stopping of EBF was short duration of maternal leave and lack of comfortable place for mothers and children at work place [15, 16].

Few of the studies recommended that workplaces should be a perfect setting for implementing policies and practices to promote and support the continuation and longer duration of EBF for employed mothers [17–19]. In workplaces of developed countries mothers have the opportunity to breastfeed their children during break time and within working hours. This helped them to successfully EBF [20]. However, in LMICs policies supporting employed mothers are uncommon [21].

Due to the above-mentioned reasons, the majority of children of employed women were more prone to childhood illness, lower bond with mothers, less immune to diseases and further expose households to substantial direct and indirect health system cost effects [5, 22, 23]. Unfavorable maternal and or child health may also negatively affect employers and national productivity growth through parental absenteeism if breastfeeding is not provided for [24]. Therefore, enabling employed women to continue breastfeeding at work place has numerous benefits for the infant, employee and organization [2, 7, 21].

Employers have an essential role in providing comfortable workplace, appropriate facilities, lactation breaks and information on relevant policies for women to feel adequately supported and encouraged to continue to breastfeed when returning to work. In the Ethiopian context, few studies assessed maternal employment as one of the determinant factors for cessation of EBF. For instance, in Halaba [25], Hawassa [16], Dilla Zuria [26] from southern Ethiopia, Bahir Dar [27], Mekele town [28] northern part, Goba and Debre Berhan city [29, 30] central Ethiopia housewives have higher odds of successful completion of EBF than employed mothers.

The government of Ethiopia has endorsed and implemented different policies and programs to reduce infant and child mortality and morbidity in the country. One of them is the Innocenti declaration, which is aimed at improving child survival through protecting, promoting and supporting breastfeeding. However, in 2017 an estimated infant mortality rate is 49.6 per 1000 live births [31]. According to GeoBase 2019 report, 63.25 per 1000 live births put the country at 14th rank among highest infant mortality rate in the world [32].

Enabling factors such as paid maternity leave, part-time work arrangements, facilities for expressing and storing breastmilk, and breastfeeding breaks for successful EBF among employed mothers were mentioned in the 2004 National strategy for Child and Young Infant Feeding in Ethiopia [33]. However, very little is known about employed mothers and determinants of EBF from employer and individual perspectives. Moreover, in Ethiopia there are limited specific studies conducted to identify determinants of EBF or reasons for cessation of EBF among permanently employed mothers in any organizations. Hence, this study investigated the prevalence and correlates of the EBF cessation among employed mothers in Dukem town, Ethiopia.

## Methods

### Study design and setting

A community based cross-sectional study was conducted in Dukem town, Oromia Special Zone Surrounding Finfine of Oromia Regional State, which is located at a distance of 37 km from Addis Ababa in the East. Dukem is one of the reform towns in the region and has a city administration municipality and four *Kebeles* (the smallest administrative unit next to the district in the Ethiopian government structure *)*. The estimated population of the town is 66,678. Out of this, 33,910 (51%) were females. The area of Dukem town is 3586 ha. Its altitude is 1800–2100 m above sea level. Dukem has economic linkages with the surrounding areas, towns, region and Addis Ababa. According to the 2014 Dukem town Labour and Social Affairs Office report, the estimated number of reproductive age women who are working in government and private sectors was around 8760. The number of mothers that had a child between 6 months and 2 years of age were 1122 from the rapid survey conducted. The study was conducted from February 28, 2015 to March 28, 2015.

### Study population

The source population was all permanently employed mothers of 6 to 24 months children working in both governmental and nongovernmental organizations in Dukem town. The study population was randomly selected from the permanently employed mothers who were working in the organizations in Dukem town and had a child between 6 months and 2 years old during the study period.

### Inclusion and exclusion criteria

A mother who completed her probation period prior to this study was included in this study. Mothers with hearing or speaking difficulty (deaf or dumb), and mothers who had infant with congenital anomalies or those who were unable to breastfeed due to illness were excluded from the study.

### Sample size and sampling technique

The required sample size for the study was determined using single-population proportion formula with the assumption of 50% prevalence of EBF cessation, 95% confidence level and 5% marginal error. The estimated sample size was 384. Since the source population (employed mothers who had a child aged between 6 months and 2 years) were 1122 (< 10,000), the finite population correction was applied and the sample size of 287 was obtained. After considering for non-response rate of 10%, the final sample size became 316**.**

**Fig. 1:**
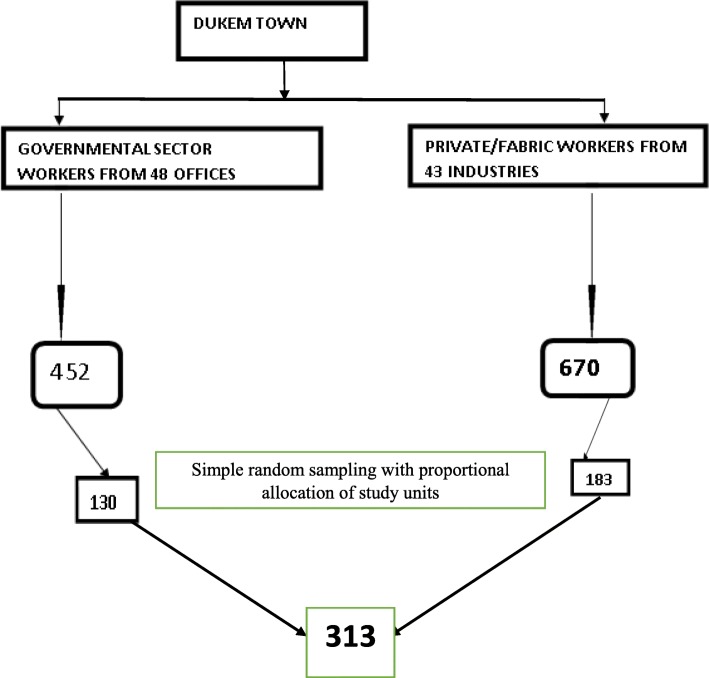
Diagrammatical illustration of sampling techniques

Mothers were selected by stratified random sampling technique. Before recruiting study participants, a quick survey was conducted to get the exact number of employed mothers who had a child between between 6 months and 2 years and working in Dukem town. Afterwards, we had identified the number of organizations, 48 governmental and 43 private. Then, the number of study participants enumeration was conducted from these organizations through rapid survey. The total number of employed mothers that had child aged between 6 months and 2 years were 1122, 452 from governmental sectors and 670 from private/fabric workers. The sample was allocated to government and private sectors using population proportion to size (PPS) technique. Computer generated random numbers were used to select mothers from each sector (private or government). *Codes* (three digit serial number and initials of organization name) assigned to each mother during rapid survey were used to contact mothers (Fig. 1). The survey tool contains name of organization, age of child and office name of mothers who had child between six months and two years.

### Study variables

#### Dependent variable

Exclusively breastfeeding cessation.

#### Independent variables

Sociodemographic/economic: Age, ethnicity, religion, educational status, marital status, monthly income, sex of child.

Work related factors: Organization support BF at work, work experience, work status, types of occupation, duration of maternity leave, duration of work, distance between workplace and child, presence of day-care center near workplace, type of employment.

### Operational and term definitions

#### EBF cessation

A mother stopping/interrupting exclusive breastfeeding her child before 6 months since delivery according to WHO recommendation for EBF (feeding only breast milk, and no other liquids or solids with the exception of oral rehydration solution, supplements or medicines to the child age less than 6 months since birth). It is a binary variable. If a mother stopped breastfeeding her child before 6 months since delivery ‘2. yes’ otherwise ‘1. no’.

#### Permanently employed mother

A mother who reported working for wages and has completed her probation period in either government or private sectors at the time of the interview.

#### Far from child

If the time to reach from workplace to child is more than 10 min on foot**.**

#### Flexible work time

Flexible working arrangements for lactating mothers which includes employment, job sharing, career break schemes, flexible hours, home-based or telework, flexible leave arrangements, leave without pay and the flexible use of annual leave.

#### Part-time work

Defined as less than 35 h per week, or a maximum of 7 h/day.

#### Reasonable lactation break

A break given to employed mothers at every work shift during the first 6 months after return to work.

#### Support for breastfeeding in the workplace

Includes several types of employee benefits, teaching employees about breastfeeding, allowing flexible scheduling to support milk expression during work, giving mothers options for returning to work such as, part-time work, provide paid and unpaid lactation break, extended maternity leave, providing onsite or near-site child care and offering professional lactation management services.

### Data collection process/measurements

Data were collected by using questionnaire adapted from the WHO, EDHS and LINKAGE project which were designed to assess infant and young child feeding practices in developing countries including Ethiopia [2, 34, 35]. In addition, some questions were developed by reviewing related literature. The questionnaire contents were work related factors (20 items), sociodemographic/economic characteristics (10 items), breastfeeding information (15 items), obstetric and health services factors (12 items) and behavioral factors (16 items). Data were collected by face-to-face interview using the questionnaire translated to local language (Afaan Oromoo).

Four diploma holder public health professionals collected the data after they were trained on research ethics particularly how to approach the study participants. Exclusive breastfeeding cessation was measured by asking eligible participants, “For how many months did they feed their child with breast milk only?”

### Data processing and analysis

Data were checked for completeness, entered into EpiData3.1, and exported to SPSS version 20 for analysis. Descriptive analysis was performed, and results presented by tables, graphs and charts. Chi-square test was performed to check adequacy of cells before performing logistic regression. Bivariable analysis was run to identify candidate variables for multivariable analysis. Variables with *p* - value ≤0.25 in bivariate logistic regression were considered as candidates for multivariable logistic regression. Multicollinearity, and interaction (using Breslow-Day Taron's test) among candidate variables were checked and none was found significant. Multivariable logistic regression was performed using backward likelihood ratio methods of variable selection to identify factors independently associated with outcome variable (i.e. cessation of EBF). Strength of association was measured using odds ratio, and 95% confidence interval. A *p* - value < 0.05 was considered statistically significant. The statistical goodness of fit for the model was checked by Hosmer and Lemeshow test.

### Data quality assurance

Two days training was given for data collectors and supervisors on the purpose of the study, questionnaire, data collection methods, and ethical concerns during data collection. Pretesting of the questionnaire was conducted on 5% of the sample size in adjacent area (Galan town) before the actual data collection. Close supervision of the data collection was carried out by the supervisors and investigator. Data were checked for completeness by supervisors and investigator on daily basis.

## Results

### Sociodemographic and socio-economic characteristics of respondents

A total of 313 mothers were included in the study. Nearly two-thirds (64.4%) were in the age group 24–29 years and the mean age of participants was 27.1 (SD = 3.44) years. Most (90.4%) of the participants were Oromoo ethnic group. Regarding the education of mothers, most (80.2%) had achieved at least diploma level. Most participants (92.6%) were married and slightly more than two-thirds (68%) had only one child. Nearly half (42.5%) earn an average monthly family income of less than or equal to 2000 Ethiopian Birr (70US$) (See Table 1).

**Table 1:** Sociodemographic characteristics of employed mothers in Dukem town

Variable	Number	Percent
Age (years)		
18–23	52	16.6
24–29	189	60.4
≥ 30	72	23.0
Ethnicity		
Oromo	283	90
Tigre	3	1.0
Amara	21	6.7
Others^a^	6	1.9
Marital status		
Single	4	1.3
Married	290	92.7
Divorced	17	5.4
Widowed	2	0.6
Educational status mothers		
Secondary	62	19.8
Diploma	138	44.1
> = Degree	113	36.1
Income		
≤ 500	4	1.3
501–1000	48	15.3
1001–1500	28	8.9
1501–2000	53	16.9
> 2001	180	57.5
Religion		
Orthodox	141	45.0
Muslim	68	21.7
Protestant	83	26.5
Others^b^	21	6.7
Family size		
≤ 3 person/HH	171	54.6
> 3 person/HH	142	45.4

^a^Waleyita, Gurage and Sidama, ^b^Waqefata, Joba

Regarding mothers working organizations 59% of study participants were employed in the private sectors or factories and 41% were employed in governmental sectors (Table 2).

**Table 2:** Work-related factors and EBF discontinuation among employed mothers in Dukem town, February-28 to March-28, 2015

Variable	Category	EBF discontinuation	
		Yes: No (%)	No: No (%)
Type of organization	Governmental	80 (61)	50 (39)
	Private/factory workers	157 (86)	26 (14)
Flexible work time	Yes	146 (69)	65 (31)
	No	91 (89)	11 (11)
Child daycare	Yes	104 (78)	29 (22)
	No	133 (74)	47 (26)
Work experience	< = 5 years	152 (79)	40 (21)
	> 5 years	85 (70)	36 (30)
Duration of work per day	Over-time	36 (100)	0 (0)
	Full time	178 (81)	42 (19)
	Part-time	23 (40)	34 (60)
Period of return to work	< = 2 months	117 (93)	9 (7)
	2–4 months	111 (70)	47 (30)
	> = 4 months	9 (31)	20 (69)
BF at workplace	Yes	18 (51)	17 (49)
	No	219 (79)	59 (21)
Time to reach from workplace to child	< 10 min	111 (72)	42 (28)
	10–20 min	78 (78)	22 (22)
	20–30 min	37 (79)	10 (21)
	> 30 min	11 (85)	2 (15)
Work overloaded	Yes	197 (78)	55 (22)
	No	40 (66)	21 (34)
Shift work	Yes	59 (92)	5 (8)
	No	178 (71)	71 (29)
Lactation break	Yes	50 (44)	63 (56)
	No	187 (93)	13 (7)

### Prevalence of exclusively breastfeeding cessation

More than three-fourths (75.7%, 95% CI: (71.0%-80.5%)) of employed mothers had discontinued exclusive breastfeeding. Among these, 80.6% discontinued EBF after the first 3 months.

### Obstetric and health service-related factors

The number of working mothers attending antenatal care (ANC) and discontinued exclusive breastfeeding were 185 (75%). Most of mothers (82.3%) delivered their infants at health facility, however, only 2% of them exclusively breastfeeding within the first 6 months of children’s age. Among those who completed postnatal care (PNC) follow-up, only 103 (33%) have EBF (Table 3).

**Table 3:** Obstetric & health service-related factors of employed mothers in Dukem town, February–March, 2015

Variable	Category	EBF discontinuation	
		Yes	No
ANC follow up	Yes	185 (75%)	62 (25%)
	No	52 (79%)	14 (21%)
Place of Delivery	Health facility	202 (75%)	68 (25%)
	Home	35 (81%)	8 (19%)
Delivery assistant	Health professional	202 (75%)	68 (25%)
	Non-health professional	35 (81%)	8 (19%)
Mode of delivery	SVD	216 (76%)	70 (24%)
	Cesarean section	21 (78%)	6 (22%)
PNC counseling	Yes	73 (71%)	30 (29%)
	No	164 (78%)	46 (22%)
Birth interval	Primiparous	105 (75%)	36 (25%)
	1–3 years	92 (79%)	25 (21%)
	> = 3 years	40 (73%)	15 (27%)
Time of initiation	< 1 h	171 (75%)	58 (25%)
	> 1 h	66 (79%)	18 (21%)

### Factors associated with EBF cessation

In bivariable logistic regression, achieving secondary education and diploma level, being employee of private organization, short duration of maternity leave, lack of a reasonable lactation break**,** being full time employee, lack of flexible working time, having shift work, work place being far from her child, not pumping breast milk, and lack of a breastfeeding place at the workplace all resulted in a significantly higher chance of discontinuing exclusive breastfeeding.

Nevertheless, in multivariable logistic regression, short duration of maternity leave (AOR 9.3; 95% CI 3.8, 23), (AOR=2.1, 95% CI(1, 4.3)), being full time employee (AOR 3.5; 95% CI 1.7, 11), lack of flexible work time (AOR 3.0; 95% CI 1.2,7.5), not pumping breast milk (AOR 4.3; 95% CI 1.7, 11), lack of a lactation break (AOR 6.7; 95% CI 3, 14.5**)** and work place being far away from her child (AOR 3.1; 95% CI 3.1, 6.3) were statistically significantly associated with the cessation exclusive breastfeeding (Table 4).

**Table 4:** Multivariable logistic regression on factors associated with EBF discontinuation among employed mothers in Dukem town, Ethiopia

Variable	EBF discontinuation		COR (95% CI)	AOR (95% CI)
	Yes: No (%)	No: No (%)		
Educational status				
Secondary	56 (90)	6 (10)	3.7 (1.5, 9.2)	–
Diploma	99 (72)	39 (28)	3.5 (1.4, 9.0)	–
Degree & above	82 (73)	31 (27)	1	
Sex of child				
Male	106 (71)	44 (29)	1	
Female	131 (80)	32 (20)	1.7 (1, 2.8)	–
Organization				
Government	80 (62)	50 (38)	1	
Private	157 (86)	26 (14)	3.8 (2.2, 6.5)	2.1 (1, 4.3)
Maternity leave				
≤ 2 months	117 (93)	9 (7)	7.3 (3.5,15.2)	9.3 (3.8, 23) *
> 2 months	120 (64)	67 (36)	1	
Intention to return to work				
Yes	195 (77)	59 (23)	1.3 (0.7, 2.5)	–
No	42 (71)	17 (29)	1	
Duration of work				
Full time	214 (84)	42 (16)	7.5 (4,14)	3.5 (1.7, 11) *
Part time	23 (40)	34 (60)	1	
Flexible work time				
Yes	146 (69)	65 (31)	1	
No	91 (89)	11 (11)	3.7 (1.8, 7.3)	3 (1.2, 7.5) *
Lactation break				
Yes	50 (44)	63 (66)	1	
No	187 (94)	13 (6)	8.7 (4.8, 15.9)	6.7 (3, 14.5) *
Shift work				
Yes	59 (92)	5 (8)	4.7 (1.8, 12.2)	–
No	178 (71)	71 (29)	1	
Far from child				
Yes	159 (81)	37 (19)	2. 2 (1.3, 3.6)	3.1 (1.5, 6.3) *
No	78 (67)	39 (33)	1	
BF at workplace				
Yes	18 (51)	17 (49)	1	
No	219 (79)	59 (21)	3.5 (1.7,7.2)	–
Pumping breast milk				
Yes	159 (71)	65 (29)	1	
No	78 (88)	11 (12)	2.9 (1.4, 5.8)	4.3 (1.7, 11) *

*p < 0.05, ‘-‘no significant association

## Discussion

The present study was carried out to investigate the prevalence and correlates of EBF cessation among employed mothers, in central part of Ethiopia. The study revealed that the prevalence of EBF cessation was 76%, which is similar with the study conducted in Northwest Ethiopia which showed EBF cessation was 79.1% among employed mothers [36]. The possible similarity might have resulted from the uniformity of policy about maternity leave for employed mothers throughout the country. Thus, the current EBF cessation among employed mothers is still high and has been a focus of maternal and child issue among employed mothers in Ethiopia.

 Several studies have found an association between maternal employment and lack of EBF in Ethiopia and elsewhere in the world. For instance, in Halaba [25], Hawassa [16], Dilla Zuria [26] from southern Ethiopia, Bahir Dar [27], Mekele town [28] northern part, Goba and DebreBerhan city [29, 30] central Ethiopia housewives have higher chance of successful completion of EBF than employed mothers. Also, studies from Angola [24], Brazil [9, 10] and Kuwait [37] showed mothers who were employed and living away from their children were more likely to discontinue EBF than non-employed mothers. However, our finding was higher than the reports from Ecuador [11] and Egypt [38]. The possible reason for the difference could be existence of policy to EBF at work place in these countries [11, 38]

The majority of mothers working in the private sector (86%) had discontinued EBF compared to mothers in the governmental organizations (62%). Those private employee mothers were two times more likely to cease EBF than governmental organization ones. Descriptively, this finding is higher than the study conducted in Malaysia, that reported EBF cessation of 57% for private versus 40% for governmental sector working mothers had discontinued EBF. However, the study didn’t show statistically significant difference between two organizations lactating mothers and ceasing EBF [39]. This might be related to the working rules and regulations differences between governmental and private organizations among the countries as well. In governmental organizations usually workers are paid on monthly salary basis, while private organizations may not tolerate employees’ absence for maternity leave.

The most frequent period of return to work from maternity leave 51%, was between the third and fourth months after birth. This finding is consistent with the study done in Brazil 52% [40]. This result is expected because maternity leave allotted in Ethiopia is only 3 months. Women who had no prolonged maternity leave may return to work immediately. Indeed, mothers may decide to stop breastfeeding early since they cannot continue to breastfeed once they returned to work. There was a great reduction of EBF cessation among mothers who returned to work after the 2 months after birth 93% when compared to 64% of those who return before 2 month age of child. Mothers who returned to work within 2 months of child’s age were about 9.3 times more likely to discontinue EBF their children than mothers who return to work after 2 months. This was supported by Biagioli F that extending maternity leave may contribute to reduction of EBF cessation [41]

As maternity leave periods typically expire before the WHO and ILO recommended period of EBF, workplaces should arrange settings for women to continue to breastfeed upon return to work. This is important to meet international and national recommendations for the health of mother and child. Important elements to support breastfeeding in the workplace would be legal provisions for paid breastfeeding breaks at workplace which enables to combine work and breastfeeding [42].

In this study, mothers who had no reasonable lactation break during working time were about 6.7 times more likely to discontinue EBF compared to their counterparts. This finding highly supported by the study from Samoa that insufficient lactation break at workplace encourages EBF cessation among employed mothers[43]. WHO and ILO recommend that employed women have a minimum of 14 weeks’ paid maternity leave and one or more daily lactation breaks or a daily reduction of hours of work to breastfeed [44].

According to the Australian Government Fair Work Ombudsman guideline, the establishment of facilities for nursing in adequate hygienic conditions at or near the workplace should be available. For an employed mother who is at work for more than 6 h per day, if she cannot take lactation breaks to breastfeed or express breast milk, her supply will diminish, and she may no longer be able to produce enough milk for her baby [45]. As a result, mothers may be forced to choose to discontinue EBF earlier than recommended period even if they do know the benefit of exclusive breastfeeding. However, there is no legally written lactation break in our country [34].

In this study, mothers who never pumped breast for their infant were 4.3 times more likely to withdraw EBF than their counterparts. Barriers of expressing milk in the work place include lack of flexibility for milk expression in the work schedule, lack of accommodations to pump or store breastmilk, lack of support from employers and colleagues, and real or perceived low milk supply. However earlier studies showed that providing employed mothers with pumping information and the necessary facilities could reduce the cessation of exclusive breastfeeding. Failing to express milk in workplace and home is a barrier against successful EBF after return to work and can lead to premature weaning [2, 17, 33, 46–49].

Inflexible working time is also another associated factor that contributes to EBF cessation. About 89% of participants, who had no flexible working time had ceased EBF. This finding is relatively similar with 84% reported in the study conducted in Taiwanese semiconductor manufacturer [50]. This could indicate that the work environment burden has negative impact on exclusive breastfeeding.

### Practical implication

Employed mothers who were working full time within 6 months after giving ***birth have higher probability of ceasing EBF*** than part time workers. This implies that working part time during the first 6 months of a child’s age, may contribute to decrease EBF cessation among employed mothers. In addition, mothers whose workplace is far from their child had more than three times the odds to discontinue EBF than those who are living close to their children.

Providing support at the workplace such as breastfeeding breaks, building and furnishing breastfeeding facilities, having flexible work time and a short duration of work should be considered to encourage EBF among employed mothers in Ethiopia. Mothers who continued EBF after returning to work need the support of their coworkers, supervisors, and others in the workplace. Individual employers can make a great effort to create a better environment that supports mothers for successful EBF, but lack of such activities discourages EBF among employed mothers [20].

However, we have tried to identify the pros and cons in this study. Conducting a rapid survey using simple random sampling techniques makes the study less prone to selection bias. Despite the strengths there were a number of drawbacks in our study. Social desirability bias could be one of these since mothers could perceive that discontinuing EBF is unacceptable for others. On the other hand, recall bias regarding EBF cessation and period of return to work may occur. Also, the HIV serostatus of mothers may confound. Mothers who have HIV virus may discontinue EBF to prevent transmission of HIV through breast milk to their child. Again, self-reporting about the EBF, age and other parameters could affect the real measurement or association between EBF cessation and predictors variables.

## Conclusion

The prevalence of EBF cessation was very high in the study area. The short duration of maternity leave, being full time worker, private organization employees, failure to pump breast milk, lack of a lactation break, inflexible work time and working far away from their child were associated with cessation of EBF among employed women. Therefore, a supportive and comfortable place should be facilitated for lactating employed mothers. There should be nationally endorsed policies to support breastfeeding mothers in all workplaces.

## Data Availability

Any data will be available from the first author Mr. Tolossa Kebede up on request.
